# Engineering *Saccharomyces cerevisiae* for targeted hydrolysis and fermentation of glucuronoxylan through CRISPR/Cas9 genome editing

**DOI:** 10.1186/s12934-024-02361-w

**Published:** 2024-03-16

**Authors:** Jonas L. Ravn, João H.C. Manfrão-Netto, Jana B. Schaubeder, Luca Torello Pianale, Stefan Spirk, Iván F. Ciklic, Cecilia Geijer

**Affiliations:** 1 Department of Life Sciences, Chalmers University of Technology, Gothenburg, 412 96 Sweden; 2 Brazilian Center for Research in Energy and Materials (CNPEM), Brazilian Biorenewables National Laboratory (LNBR), Campinas, 13083-100 Brazil; 3 Institute of Bioproducts and Paper Technology (BPTI), Graz University of Technology, Inffeldgasse 23, Graz, 8010 Austria; 4 Estación Experimental Agropecuaria Mendoza, Instituto Nacional de Tecnología Agropecuaria (INTA), 5507 Luján de Cuyo, San Martín, Mendoza, 3853 Argentina

**Keywords:** Yeast, Xylan, Xylanase, α-glucuronidase, Metabolic engineering, Co-culture, Microbial cell factories, Consolidated bioprocessing

## Abstract

**Background:**

The abundance of glucuronoxylan (GX) in agricultural and forestry residual side streams positions it as a promising feedstock for microbial conversion into valuable compounds. By engineering strains of the widely employed cell factory *Saccharomyces cerevisiae* with the ability to directly hydrolyze and ferment GX polymers, we can avoid the need for harsh chemical pretreatments and costly enzymatic hydrolysis steps prior to fermentation. However, for an economically viable bioproduction process, the engineered strains must efficiently express and secrete enzymes that act in synergy to hydrolyze the targeted polymers.

**Results:**

The aim of this study was to equip the xylose-fermenting *S. cerevisiae* strain CEN.PK XXX with xylanolytic enzymes targeting beechwood GX. Using a targeted enzyme approach, we matched hydrolytic enzyme activities to the chemical features of the GX substrate and determined that besides endo-1,4-β-xylanase and β-xylosidase activities, α-methyl-glucuronidase activity was of great importance for GX hydrolysis and yeast growth. We also created a library of strains expressing different combinations of enzymes, and screened for yeast strains that could express and secrete the enzymes and metabolize the GX hydrolysis products efficiently. While strains engineered with *Bm*Xyn11A xylanase and XylA β-xylosidase could grow relatively well in beechwood GX, strains further engineered with Agu115 α-methyl-glucuronidase did not display an additional growth benefit, likely due to inefficient expression and secretion of this enzyme. Co-cultures of strains expressing complementary enzymes as well as external enzyme supplementation boosted yeast growth and ethanol fermentation of GX, and ethanol titers reached a maximum of 1.33 g L^− 1^ after 48 h under oxygen limited condition in bioreactor fermentations.

**Conclusion:**

This work underscored the importance of identifying an optimal enzyme combination for successful engineering of *S. cerevisiae* strains that can hydrolyze and assimilate GX. The enzymes must exhibit high and balanced activities, be compatible with the yeast’s expression and secretion system, and the nature of the hydrolysis products must be such that they can be taken up and metabolized by the yeast. The engineered strains, particularly when co-cultivated, display robust growth and fermentation of GX, and represent a significant step forward towards a sustainable and cost-effective bioprocessing of GX-rich biomass. They also provide valuable insights for future strain and process development targets.

**Supplementary Information:**

The online version contains supplementary material available at 10.1186/s12934-024-02361-w.

## Introduction

Glucuronoxylan (GX) is a major hemicellulose in several agricultural and forestry side- and waste streams, which can serve as starting material for production of value-added products using yeast cell factories [1]. GX polymers present in hardwoods such as birch and beech are composed of a backbone of β-1,4-linked xylopyranosyl units often *O*-acylated at the C-2 or C3- positions and substituted with α-1,2- linked (methyl)-glucuronic acid residues and/or occasionally α-1,2- or α-1,3-linked arabinosyl units [2]. Given that most yeasts only metabolize monosaccharides and disaccharides, the GX polymers must be hydrolyzed through pretreatment and enzymatic hydrolysis before they can be used as a carbon source [3, 4]. However, pretreatments such as acid hydrolysis produce compounds that inhibit the fermenting cell factories, and supplementation of enzymes for hydrolysis adds substantially to the production cost [5]. As an alternative, GX-degrading yeasts can be used in consolidated bioprocessing where production of enzymes, polymer hydrolysis and fermentation occur simultaneously, which has the potential to reduce costs and simplify the production process [6]. Natural GX-degrading yeasts exist in nature [7, 8], however these yeasts are not well characterized and genetic tools for these species are often missing, making strain and process development ineffective. Instead, the well characterized baker’s yeast *Saccharomyces cerevisiae* can be equipped with enzymes for hydrolysis and conversion of GX into bioproducts of interest [9–11].

*S. cerevisiae* cannot naturally metabolize xylose, the most abundant monomeric saccharide in GX. Extensive metabolic engineering efforts have resulted in multiple *S. cerevisiae* strains that readily ferment xylose into ethanol under oxygen limited conditions [12, 13], and a few of these strains have been further equipped with enzymes for xylan degradation [14, 15]. Overall, the strains developed so far display relatively slow growth in xylan and low ethanol titers (summarized in Supplemental Table S1). There are several possible reasons for the inefficient conversion of this non-conventional and complex carbon source. A majority of the studies use plasmid-based gene expression rather than stable gene integration in the yeast genome, which often results in low and unbalanced expression of the enzymes [16]. Haploid, laboratory auxotrophic strains are also commonly used, likely due to the ease of transformation and availability of selectable markers, although they generally display slower growth and lower fermentative capacity compared to diploid or even polyploid prototrophic strains of industrial origin [17, 18]. Moreover, the relatively low capacity of *S. cerevisiae* to secrete enzymes [19] poses a significant bottleneck, as efficient enzyme secretion is a prerequisite for the hydrolytic enzymes to come in close physical proximity to the GX polymers that are too large to traverse the yeast cell wall and to be taken up by the cell. All these issues must be solved before the strains can be used for industrial applications.

Designing an efficient GX-degrading yeast also includes identifying and expressing the optimal enzymes for the purpose. The enzymes must be compatible both with the expression and secretion systems of *S. cerevisiae* and display high and balanced activities and act in synergy to efficiently break down GX under varying conditions. Moreover, the nature of the hydrolysis products must be such that they can be taken up and metabolized by the yeast. Several microorganisms from diverse kingdoms that specialize in catabolizing GX have been discovered and described, which offers diverse enzymatic strategies and gene targets for metabolic engineering of *S. cerevisiae* [20–22]. However, despite the large optimization possibilities of screening several different enzymes, most engineering approaches to date have employed β-xylosidases and endo-1,4-β-xylanases from very similar enzyme families (Supplemental Table S1). A less studied strategy in depolymerization of GX involves targeting the major sidechains in GX such as (4-*O*-methyl)-D-glucuronic acid, L-arabinose (although found mainly in softwood glucurono-arabinoxylan) and the acetyl groups using de-branching enzymes. Removal of these sidechains enables more cleavage sites for xylanases [23], ultimately leading to more available metabolizable xylose and higher conversion titers and yields.

Taking all the above-mentioned parameters into consideration, the aim of this study was to engineer *S. cerevisiae* for tailored and efficient GX hydrolysis and fermentation. Here we employed *S. cerevisiae* CEN.PK XXX, a diploid, prototrophic strain equipped with xylose reductase (*XYL1*), xylitol dehydrogenase (*XYL2*) and xylulokinase (*XKS1*) genes for xylose growth and fermentation [24]. Based on a targeted enzyme approach, a library of expression cassettes with genes encoding different xylanolytic enzymes that specifically target beechwood GX and its sidechains was constructed and integrated in the yeast genome using CRISPR/Cas9 genome editing tools. The engineered strains showed a range of different enzymatic activity levels and growth and fermentation capacities, where co-cultures of strains with complementing enzymes clearly outcompeted monocultures. The results can guide future strain development towards cell factory design for efficient consolidated bioprocessing.

## Materials and methods

### Enzymatic treatment of thin films and SPR

Cellulose thin films (∼ 30 nm) obtained by regeneration of trimethylsilyl cellulose (TITK, Germany) [25] were coated with birchwood GX (Sigma, Germany) as described previously [26], resulting in a xylan layer thickness of ∼ 20 nm. Enzyme solutions of endo-1,4-β-xylanases from *Cellvibrio japonicus Cj*GH10 (cat. no. E-XYNACJ, Megazyme, Ireland), *Bacteroiodes ovatus Bo*GH30 (Industrial Biotechnology, Chalmers, Sweden) and *Blastobotrys mokoenaii Bm*Xyn11A GH11 (Ravn et al. 2023, Industrial Biotechnology, Chalmers, Sweden) were diluted to 5 U⋅mL^− 1^ in 100 mM sodium phosphate buffer, pH 5 and with 0.5 mg⋅mL^− 1^ bovine serum albumin acting as protein stabilizing agent. The degradation experiments were performed with a MP-SPR Navi™ 210 VASA from BioNavis Ltd. (Finland) using a 785 nm laser. All measurements were carried out at 25 °C using an angular scan range of 50 to 78° and a scan speed of 8 °/s. The coated SPR sensor slides (glass substrate with a 5 nm thick chromium adhesion layer and a 50 nm thick gold layer) were equilibrated in sodium phosphate buffer (100 mM, pH 5) containing 0.5 mg⋅ml^− 1^ BSA for about 30 min at a flow rate of 25 µl⋅min^− 1^. After equilibration, the endo-1,4-β-xylanase solution was injected into the SPR chamber for 8 min at a flow rate of 25 µl⋅min^− 1^, followed by a 30 min rinsing step. A total of 200 µl enzyme solution was applied on the thin films. Triplicates were performed for each experiment. BioNavis Dataviewer software was used for data processing. The De Feijter equation (Eq. 1) [27] was used to calculate the amount of degraded xylan (mg⋅m^− 2^). The change in SPR angle ΔΘ (°) was calculated by subtracting the average stabilized SPR angle after the experiment (10 min) from the average stabilized SPR angle before the experiment (10 min). The term k⋅d_p_ (cm/°) can be considered constant for thin films < 100 nm and is 1.90⋅ 10^− 7^ cm/° for the 785 nm laser in aqueous systems for the used SPR instrument. A refractive index increment (dn/dc) of 0.158 cm^3^⋅g^− 1^ was used as determined in an earlier study [26]. To convert the decrease in SPR angle to a reduction in layer thickness a xylan density of 1.2 g⋅cm^3^ was assumed.

1 $${\Gamma }=\frac{{\Delta }{\Theta } \text{k} {\text{d}}_{\text{p}}}{dn/dc}$$

### Construction of plasmids

All plasmids were assembled using the MoClo Modular Cloning System Plasmid Kit [28] and the ScEnSor Kit [29]. Heterologous genes include a GH3 XylA β-xylosidase from *Aspergillus oryzae* KBN616 [30], three GH11 endo-1,4-β-xylanases: *Bm*Xyn11A from the yeast *Blastobotrys mokoenaii* CBS 8435 [22], GH11 XynHB from *Bacillus sp.* HBP8 [31], GH11 XynB from *A. niger* CBS 513.88 [32] and a GH115 α-methyl-glucuronidase Agu115 from *Schizophyllum commune* H4-8 FGSC 9210 [33, 34]. Codon-optimized genes with overhangs 5’GCATCGTCTCATCGGTCTCATTCTTT3’ and 5’TTATCCTGAGACCTGAGACGGCAT3’ for BsaI restriction digestion and ligation into LT1_30_backbone_X2 backbone plasmid were ordered from GeneScript (USA). The final plasmid constructs including promoters (constitutive), terminators and the secretory signal peptide *SED1* from *S. cerevisiae* fused to the N-terminal of the target genes are listed in Table 1. Details on plasmid assemblies with promoters, terminators, the *SED1* signal peptide and primers can be found in Supplementary Table S2. Moreover, a schematic overview of the assembled plasmids and the codon optimized sequences for the target genes can be found in Supplementary File S1.

Competent *Escherichia coli* DH5α used for plasmid construction and amplification was grown in Luria-Bertani medium (1% (w/ v) tryptone, 0.5% (w/v) yeast extract, and 0.5% (w/v) sodium chloride) containing the required antibiotic (chloramphenicol 25 µg/mL, ampicillin 100 µg/mL or neomycin 50 µg/mL).

**Table 1:** Plasmids constructed and used in this study

Plasmid Full Name	Description	Source
pJR1_01_SED1-XylA	TU1 that expresses *Aspergillus oryzae* SED1-XylA β-xylosidase under ScPGK1 promoter and ScTDH1 terminator with SED1 signal peptide (N-term)	This work
pJR1_02_SED1-BmXyn11A	TU1 that expresses *Blastobotrys mokoenaii* SED1-BmXyn11A xylanase under ScTDH3 promoter and ScPGK1 terminator with SED1 signal peptide (N-term)	This work
pJR1_03_SED1-XynHB	TU1 that expresses *Bacillus sp.* HBP8 SED1-XynHB xylanase under ScTDH3 promoter and ScPGK1 terminator with SED1 signal peptide	This work
pJR1_04_SED1-XynB	TU1 that expresses *Aspergillus niger* SED1-XynB xylanase under ScTDH3 promoter and ScPGK1 terminator with SED1 signal peptide	This work
pJR1_08_SED1-Agu115	TU1 that expresses *Schizophyllum commune* SED1-Agu115 α-glucuronidase under ScCCW12 promoter and ScADH1 terminator with SED1 signal peptide (N-term)	This work
pJR2_01_SED1-XylA-BmXyn11A	TU2 expressing genes from JR1_01 and JR1_02	This work
pJR2_02_SED1-XylA-XynHB	TU2 expressing genes from JR1_01 and JR1_03	This work
pJR2_03_SED1-XylA-XynB	TU2 expressing genes from JR1_01 and JR1_04	This work
pJR2_04_SED1-XylA-BmXyn11A-Agu115	TU3 expressing genes from JR1_01, JR1_02 and JR1_08	This work
pJR2_05_SED1-XylA-XynHB-Agu115	TU3 expressing genes from JR1_01, JR1_03 and JR1_08	This work
pJR2_06_SED1-XylA-XynB-Agu115	TU3 expressing genes from JR1_01, JR1_04 and JR1_08	This work

### Yeast transformation

Yeast transformation was performed using the ScEnSor Kit [29] based on the LiAc/salmon sperm carrier DNA with polyethylene glycol method [35]. The CRISPR/Cas9 targeted the X2 locus site in chr. X [36, 37] with guide RNA LT58_sgRNA1_X2 sequence 5’TGCATAATCGGCCCTCACAG3’. A step-by-step guide for cloning and transformation was used from the ScEnSor Kit [29]. Yeast strains were pre-cultured in 1% Yeast Extract, 2% Peptone, 2% Dextrose (YPD) medium and strains were engineered with single or combinations of multiple genes encoding β-xylosidases, endo-1,4-β-xylanases and α-methyl-glucuronidases as listed in Table 2. Colony PCR was performed to confirm genomic integration of recombinant gene cassettes with primers annealing to the genome adjacent to integration X2 site (Supplementary Figure S2).

**Table 2:** Strains constructed and used in this study

Strain	Abbreviated name	Description	Source
*S. cerevisiae* CEN.PK XXX	XXX	Expresses *RPE1*, *TAL1*, *RKI1* and *XKS1* genes, and insertion of codon optimized *XYL1* and *XYL2* genes from *Scheffersomyces stipitis* into the genome of parental strain *S. cerevisiae* CEN.PK 122 MDS	Westman et al. 2014
*S. cerevisiae* CEN.PK XXX-SED1.XylA	XylA	*S. cerevisiae* with secreted β-xylosidase activity	This work
*S. cerevisiae* CEN.PK XXX-SED1.BmXyn11A	BmXyn11A	*S. cerevisiae* with secreted xylanase activity	This work
*S. cerevisiae* CEN.PK XXX-SED1.XynHB	XynHB	*S. cerevisiae* with secreted xylanase activity	This work
*S. cerevisiae* CEN.PK XXX-SED1.XynB	XynB	*S. cerevisiae* with secreted xylanase activity	This work
*S. cerevisiae* CEN.PK XXX-SED1.Agu115	Agu115	*S. cerevisiae* with secreted α-methyl-glucuronidase activity	This work
*S. cerevisiae* CEN.PK XXX-SED1.XylA-SED1.BmXyn11A	XylA-BmXyn11A	*S. cerevisiae* with secreted β-xylosidase and xylanase activity	This work
*S. cerevisiae* CEN.PK XXX-SED1.XylA-SED1.XynHB	XylA-XynHB	*S. cerevisiae* with secreted β-xylosidase and xylanase activity	This work
*S. cerevisiae* CEN.PK XXX-SED1.XylA-SED1.XynB	XylA-XynB	*S. cerevisiae* with secreted β-xylosidase and xylanase activity	This work
*S. cerevisiae* CEN.PK XXX-SED1.XylA-SED1.BmXyn11A- SED1.Agu115	XylA-BmXyn11A-Agu115	*S. cerevisiae* with secreted β-xylosidase, xylanase and α-methyl-glucuronidase activity	This work
*S. cerevisiae* CEN.PK XXX-SED1.XylA-SED1.XynHB-SED1.Agu115	XylA-XynHB-Agu115	*S. cerevisiae* with secreted β-xylosidase, xylanase and α-methyl-glucuronidase activity	This work
*S. cerevisiae* CEN.PK XXX-SED1.XylA-SED1.XynB- SED1.Agu115	XylA-XynB-Agu115	*S. cerevisiae* with secreted β-xylosidase, xylanase and α-methyl-glucuronidase activity	This work

### Enzyme activity of CRISPR/Cas9 engineered strains

Engineered strains were assayed for secreted endo-1,4-β-xylanase activity using a 200 µL mixture of 10 g L^− 1^ beechwood GX (Megazyme, Ireland, monosaccharides composition (%): xylose: glucuronic acid: other sugars = 86.1: 11.3: 2.6), in 50 mM sodium acetate buffer (pH 5.5) using 50 µL cell-free supernatant from 15 mL overnight YPD yeast cultures (OD ∼ 7). The mixture was incubated for 30 min at 40 °C at 400 rpm followed by immediate chilling on ice and inactivation at 98 °C for 5 min. Reduced sugar ends were determined using the dinitrosalicylic acid (DNS) method [38]. One unit of enzyme activity was defined as the amount of enzyme required to release 1 µmol of reducing saccharides in 1 min under the assay conditions.

For β-xylosidase subcellular activities (secretome, cell-associated and intracellular) cell-free supernatant or milliQ washed cell pellets (cell OD_600_ = 3) was incubated with 5 mM *p*-nitrophenyl-β-D-xylopyranoside in 200 µL reactions in a 96-well plate containing 20 mM sodium phosphate (pH 7) for 30 min at 30 °C at 400 rpm. Intact cells were removed by centrifugation (4,000 x g, 5 min) and 100 µL was transferred to a new 96-well plate for *p*-nitrophenol quantification at 405 nm. The intracellular fraction (cell OD_600_ = 3) was lysed by eight cycles of bead beating at 8,000 rpm, 30 s, followed by the addition of Y-PER (yeast protein extraction reagent; Pierce, Rockford, IL, USA). The soluble intracellular fraction was isolated by centrifugation (13,000 x g, 5 min) and assayed alongside the cell-free supernatant (secretome) and the intact cell pellets.

Subcellular (secretome, cell-associated and intracellular) α-methyl-glucuronidase activity was determined using the NADH-based D-glucuronic acid kit from Megazyme (Ireland). An assay mixture of 54 µL from a 200 µL incubation of 10 g L^− 1^ beechwood GX (Megazyme, Ireland) in 50 mM sodium acetate buffer (pH 5.5) containing 50 µL of intracellular fraction incubated at 40 °C for 30 min at 400 rpm was quantified at 340 nm. Secretome, intact cells and intracellular fractions were prepared as described above.

### Growth characterization in glucuronoxylan

The XXX strain and CRISPR/Cas9 engineered strains were pre-cultured in 2 mL YPD O/N at 30 °C, 200 rpm and harvested by centrifugation (4500 rpm, 5 min). Cells were washed twice in MQ and inoculated in 250 µL Delft medium (pH 5) + 2 g L^− 1^ beechwood GX (Megazyme, Ireland) at a starting OD_600_ = 0.1 in a 96 well format. In co-cultures, a strain ratio of 1:1 was used. For enzyme supplementation tests, endo-1,4-β-xylanase *Bm*Xyn11A [22], β-xylosidase *Sr*GH43 from *Selenomonas ruminantium* (cat. no. E-BXSR; GH43, Megazyme, Ireland), α-methyl-glucuronidase *Bo*Agu115A from *Bacteriodes ovatus* (cat. no. CZO311; GH115, NZYTech (Portugal) and acetyl xylan esterase *Os*CE6 from *Orpinomyces sp.* (cat. no. E-AXEAO; CE6 Megazyme, Ireland) were added at a concentration of 200 µg/g GX. Growth was monitored over time at 30 °C and 200 rpm, and all yeast strains were grown in triplicates in a 96-well plate setup in a Growth-Profiler 960 (EnzyScreen, Netherlands). Growth rates were calculated using the PRECOG online tool, found at http://precog.lundberg.gu.se/ [39].

For agar plate assays, washed yeast precultures (10mL YPD) were diluted in MiliQ water and a 10 µL drop of OD_600_ = 5 were pipetted onto Delft minimal medium agar plates (2%) containing 0.8% beechwood GX (Megazyme, Ireland). Plates were incubated at 30 °C for 10 days, and pictures were taken daily to follow xylan clearing zones and yeast colony growth. Picture brightness was edited using the Affinity Photo 2 software.

### Co-culture fermentations of GX with enzyme supplementation in Erlenmeyer flasks

Metabolite formation was determined in co-culture fermentations using 25 mL synthetic Delft medium (pH 5) containing 0.79 g L^− 1^ CSM complete supplement mixture (MP biomedicals, USA) and either 20 g L^− 1^ xylose, xylooligosaccharides (XOs) from corn cob (> 95% XOs, Roth, Germany) or beechwood GX (Megazyme, Ireland). Moreover, the effect of enzyme supplementation on metabolite formation was compared by adding *Bm*Xyn11A and *Bo*Agu115A with enzyme concentration of 100 µg enzyme/g GX. The strains were pre-cultured individually in YPD over night at 30 °C, 200 rpm, centrifuged (4500 rpm, 8 min), washed in MQ and then harvested by another centrifugation. Aliquots of the strains were combined in co-cultures with a 1:1 ratio and an initial OD_600_ = 5 for each strain, and let to ferment at 30 °C, 200 rpm for 48 h. All fermentations were performed in triplicates in 100 mL baffled Erlenmeyer flasks with glycerol airlock systems that allow CO_2_ outflow and prevent O_2_ inflow and thereby creating an oxygen limited environment.

### Batch co-culture fermentations and metabolite analysis in bioreactors

Batch fermentations were carried out under controlled conditions in 1 L DASGIP Bioreactors (Juelich, Germany) containing an initial working volume of 300 mL for co-cultures of XylA + XylA-BmXyn11A or XylA + XylA-BmXyn11A-Agu115 in 1:1 strain ratio. Fermentations were performed in synthetic Delft medium with 0.79 g L^− 1^ CSM complete supplement mixture (MP biomedicals, USA) and 20 g L^− 1^ beechwood GX and maintained at pH 5 with 2 M KOH. An initial OD_600_ = 5 for each strain was used with constant stirring of 400 rpm at 30 °C and 5% aeration (1,05% O_2_) 1 mL of a 2% (v/v) antifoam solution (Antifoam 204; Sigma Aldrich, USA) and addition of recombinant *Bm*Xyn11A (in house, Chalmers) and *Bo*Agu115A (NZYTech, Portugal) enzymes at 100 and 50 µg/g GX, respectively.

Culture samples (duplicates or triplicates) were filtered through 0.2-µm nylon membrane filters (VWR, USA) and analyzed using high performance liquid chromatography. The concentration of xylose, xylitol and ethanol from Delft synthetic medium with 20 g L^− 1^ Beechwood GX media was determined using a Dionex UltiMate 3000 series HPLC (ThermoFisher Scientific, USA) equipped with a Dionex RI-101 refractive index detector and an Aminex HPX-87 H column (7.8 × 300 mm, Bio-Rad, USA) operating at 50 °C and 0.7 mL/min of a flow rate with 5 mM H_2_SO_4_ as an isocratic mobile phase.

## Results

### Characterization and selection of xylanases

**Fig. 1:**
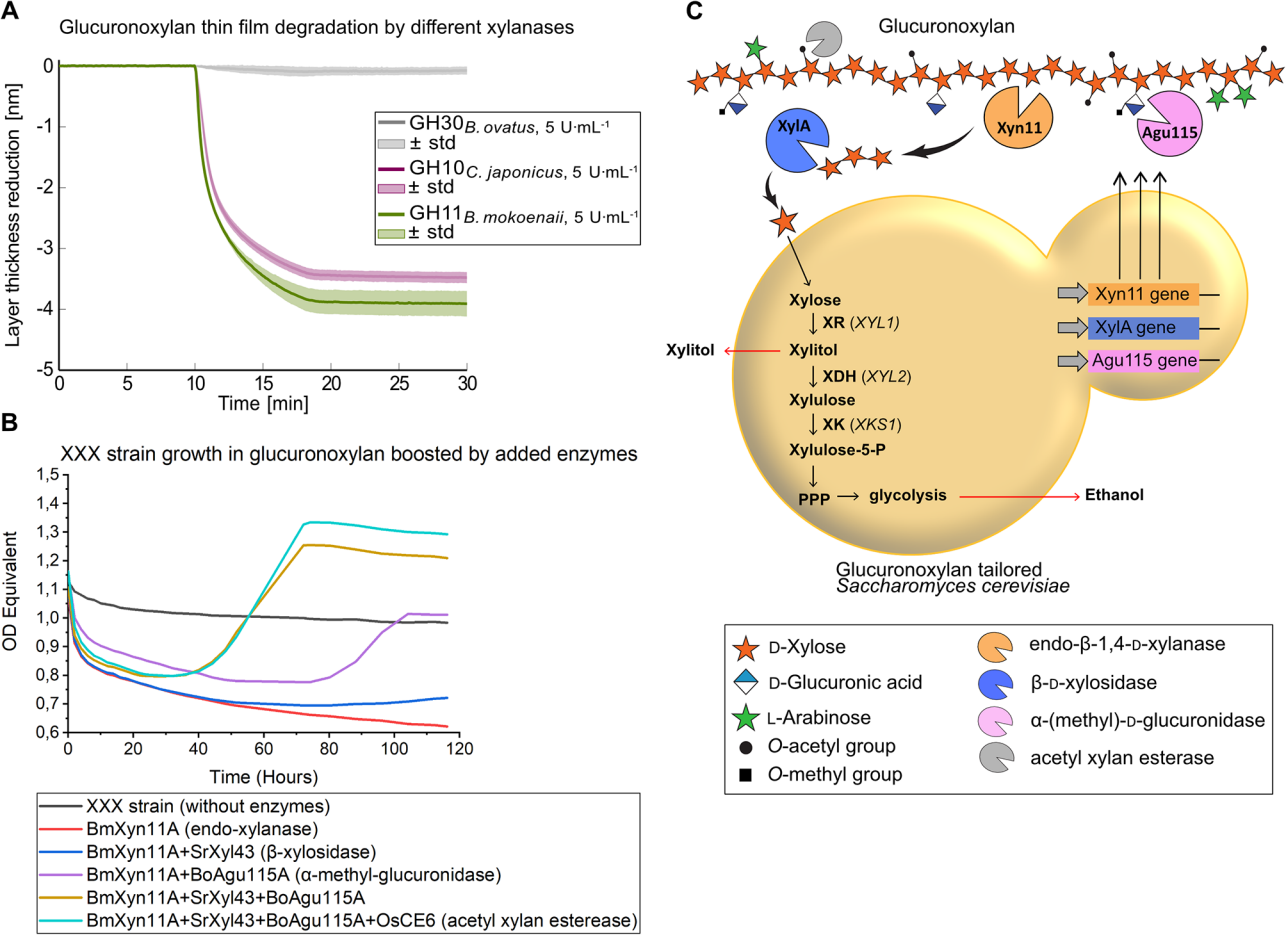
Enzymatic activities in relation to *S. cerevisiae* growth on glucuronoxylan. (**A**) Comparison of the capacity of different xylanases to degrade GX on cellulose thin films, as quantified by a decrease in film layer thickness after treatment with sodium phosphate buffer for 10 min until application of xylanase to the film performed in biological triplicates. (**B**) Growth performance of the xylose-fermenting *S. cerevisiae* XXX strain over time when supplemented with different combinations of xylanase, β-xylosidase, α-methyl-glucuronidase and acetyl xylan esterase performed in biological triplicates. (**C**) Overview of genes and recombinant enzymes (Xyn11, XylA and Agu115) involved in GX depolymerization and the xylose metabolic pathway of the engineered XXX strain. OD Equivalent = Optical density normalized from *S. cerevisiae* growth in Delft/glucose medium in a Growth-Profiler 960. GH = glycoside hydrolase. GX = glucuronoxylan.

To engineer the xylose-fermenting *S. cerevisiae* CEN.PK XXX strain for efficient hydrolysis and conversion of polymeric beechwood GX, a suitable enzyme arsenal is required. Endo-1,4-β-xylanases are the principal enzymes needed for GX depolymerization, and these enzymes are found in different Glycoside Hydrolase (GH) families with diverse structures and functions. To find the most suitable xylanase for GX depolymerization, we screened xylanases from GH family 10, 11 and 30 for activity against cellulose thin films coated with GX, which emulates native cellulose-xylan interactions in xylan-rich biomass substrates [26]. Xylanases from these three GH families were selected, as they are known to target and cleave the GX xylan backbone at different moieties depending on the degree of branching [40]. As GX is enzymatically degraded, the GX layer thickness decreases which is reflected by a decrease in the ∆SPR angle, and the thin films represent a real time and sensitive assay to screen xylanase activity. Here, the *Bm*Xyn11A GH11 xylanase from the yeast *B. mokoenaii*, identified and purified in one of our previous studies [22], showed a faster degradation than the GH10 from *C. japonicus* and GH30 from *B. ovatus*, as indicated by the steeper drop in the curve from minute 8 (Fig. 1A). A layer thickness reduction of -3.9 ± 0.3 nm was achieved with the *Bm*Xyn11A GH11 xylanase, while a layer thickness reduction of -3.5 ± 0.3 nm was determined for the GH10 from *C. japonicus* and a reduction of only − 0.08 ± 0.06 nm for the GH30 from *B. ovatus*. GH11 xylanases are well-described for their characteristic and conserved “thumb-loop” β-jelly-roll structure/function providing them high xylan catalytic efficiency, and their small sizes (∼ 20 kDa) and broad pH and temperature optima make these xylanases suitable for many biotechnological applications [41]. These aspects, together with the positive thin film results, led us to select the GH11 xylanase family for further studies.

To ensure that the yeast can make use of the GX degradation products as carbon and energy sources, we grew the xylose-fermenting XXX strain in minimal medium containing 20 g L^− 1^ beechwood GX in the presence of externally added enzymes in different combinations (Fig. 1B). Addition of only the *Bm*Xyn11A xylanase resulted in a decrease in OD_Equ_, which is due to solubilization of partly insoluble GX. Addition of *Bm*Xyn11A together with GH43 β-xylosidase from *S. ruminantium* resulted in slow but detectable growth, whereas these enzymes in combination with the debranching enzyme GH115 α-methyl-glucuronidase from *S. commune* enabled yeast to grow with a doubling time of 7.2 h during the exponential phase and a final OD_Equ_ of 1.2. Additional de-acylation activity by a supplementation of a carbohydrate esterase (CE) family 6 enzyme from *Orpinomyces sp.* had a less drastic effect on growth and resulted in a doubling time of 6.4 h in the exponential phase and a final OD_Equ_ of 1.3 (Fig. 1B). Combined, these results suggest that expression of enzymes targeting the two major chemical features of GX, the β-1,4-xylan backbone and the 4-*O*-methyl-D-glucuronic acid sidechains, would result in an efficient GX-converting strain. As the acetyl xylan esterase CE6 contribution was modest, it was not pursued further for genomic engineering in this study. The chosen strain engineering design can be viewed in Fig. 1C.

### Recombinant strain development and heterologous enzyme activity assays

**Fig. 2:**
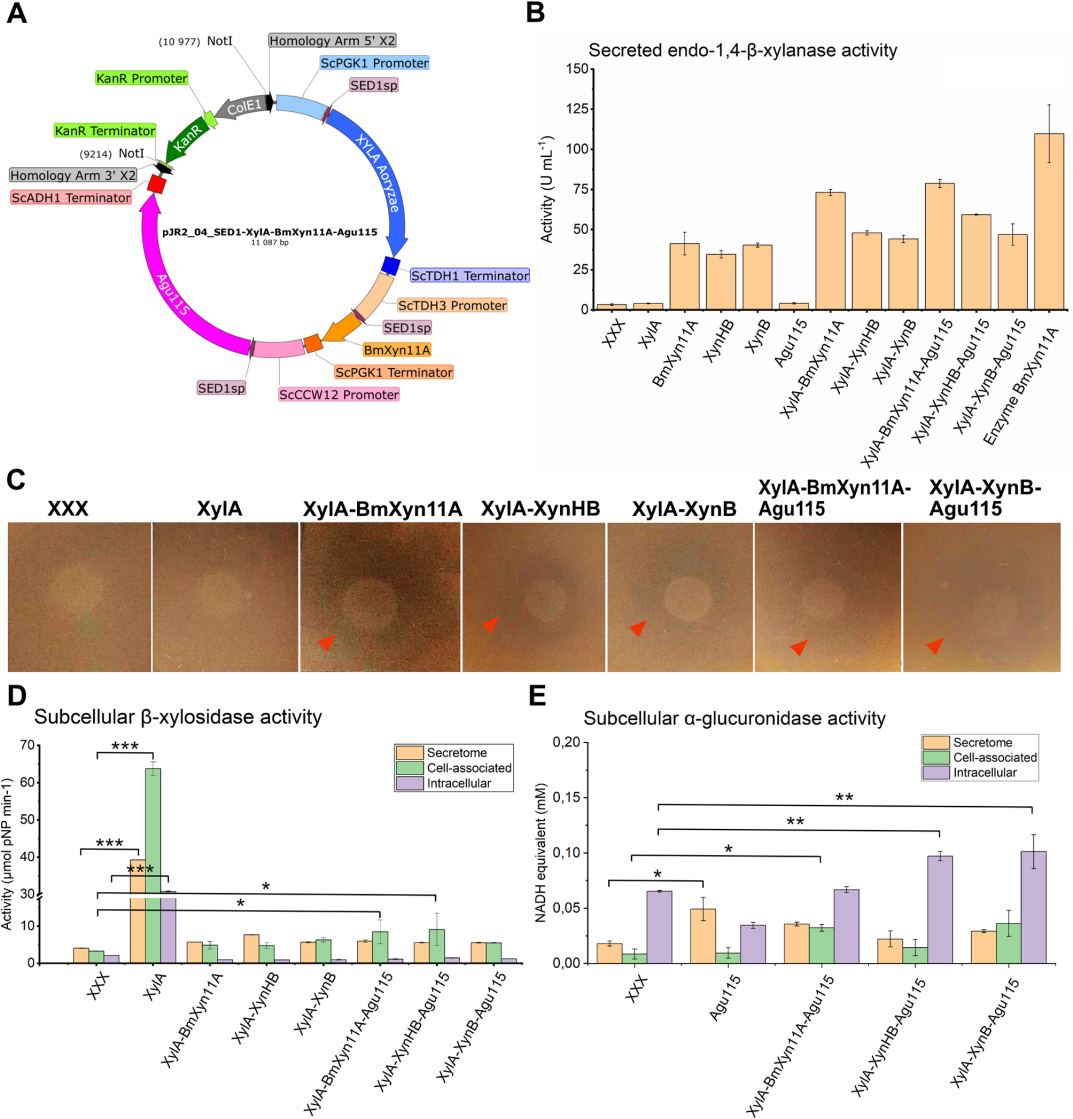
Xylanolytic activities of yeast strains engineered with CRISPR/Cas9. (**A**) Schematic map of the plasmid pJR2_04_SED1-XylA-BmXyn11A-Agu115 containing XylA β-xylosidase, *Bm*Xyn11A xylanase and Agu115 α-methyl-glucuronidase genes with NotI plasmid linearization sites adjacent to homology arms for homologous recombination into the *S. cerevisiae* genome at the X2 locus. (**B**) Secreted xylanase activity from cell-free supernatants of yeast strains grown in 2 mL YPD and incubated in 10 g L^− 1^ beechwood GX compared to purified recombinant 1 mg mL^− 1^*Bm*Xyn11A using DNS reducing sugar assays in triplicates. Strain names indicate what recombinant enzyme is engineered e.g. xylanase (BmXyn11A, XynHB or XynB). (**C**) Clearing zones (indicated by red arrows) on agar plates containing Delft medium with 8 g L^− 1^ beechwood GX mediated by heterologous xylanase secretion from co-expression strains after 48 h incubation at 30 °C using a 10 µL drop with OD = 5 cell density. (**D**) Subcellular β-xylosidase activity quantified using *p*-nitrophenyl-β-D-xylopyranoside and (**E**) subcellular α-methyl-glucuronidase activity determined by NADH-based D-glucuronic acid in duplicates. Values are means ± standard deviations as error bars. Asterisks indicate statistical significance in subcellular activity levels between the XXX strain and engineered strains. *P* values ≤ 0.05 (*), ≤ 0.01 (**) and ≤ 0.001 (***) were considered significant (*n* = 2–3) and evaluated using one-way ANOVA Dunnet’s test with XXX fractions as control group

For yeast strain engineering, plasmids with single or multiple translational units for genomic integration in *S. cerevisiae* were developed using the ScEnSor Kit [29], exemplified in Fig. 2A. As xylanase activity is key for xylan degradation and knowing that both expression and secretion efficiency can differ between recombinant enzymes, we expressed three GH11 xylanases from different kingdoms (yeast, fungal and bacterial): the *B. mokoenaii* yeast-derived *Bm*Xyn11A assessed in the thin film experiment as well as a fungal XynB from *A. niger* and a bacterial XynHB from *Bacillus sp.* HBP8 with previously reported high activities in GX hydrolysis [31, 32]. Furthermore, a GH3 XylA β-xylosidase from *A. oryzae* and a GH115 (Agu115) α-methyl-glucuronidase from *S. commune* were engineered. As it can be challenging for *S. cerevisiae* to secrete multiple recombinant proteins [42], a library of strains expressing single enzymes (XylA, BmXyn11A, XynB, XynHB or Agu115) and combinations of enzymes was generated. All enzymes were fused to the *Sed1* signal peptide that directs proteins to the extracellular space, enabling comparison of enzyme expression and activity in the secretome [43] (Fig. 2A).

To determine if the constructed yeast strains expressed and secreted functional enzymes, enzyme activity assays were performed on secretomes of strains grown overnight in YPD medium. All strains expressing xylanases exhibited significant secreted activities compared to the negative control. Notably, strains expressing *Bm*Xyn11A consistently demonstrated higher activities compared to those expressing XynHB and XynB, with values ranging from 41-79 U mL^− 1^ compared to 34–59 U mL^− 1^, and 40–47 U mL^− 1^, respectively (Fig. 2B).

Additionally, a presence of clearing zones surrounding the yeasts grown on semi-solid GX agar plates was observed, indicating xylan degradation attributable to the activities of secreted xylanases (Fig. 2C).

For β-xylosidase activity, the strain engineered with a single XylA gene showed significantly higher activity compared to strains co-expressing the β-xylosidase with other enzymes. To assess whether the lower β-xylosidase activities in the latter secretomes are due to enzymes being trapped intracellularly or within the cell wall, we also performed enzyme assays on intact and dispersed cells. However, the relatively low activity detected in all fractions indicates that the enzyme expression and/or activity is negatively affected by co-expression with other heterologous enzymes (Fig. 2D). In opposite, co-expression strains engineered with the Agu115 α-methyl-glucuronidase showed higher activity in both the cell-attached and intracellular fractions than in the secretome, indicating that a large fraction of the 107 kDa enzyme was retained inside or close to the cell. Moreover, strains co-expressing Agu115 and a xylanase showed increased α-methyl-glucuronidase activity compared to strains without the xylanase (Fig. 2E), which correlates well with previous findings that GH115 α-glucuronidase activity increases when acting in synergy with a xylanase [34]. From these experiments we can conclude that the engineered strains express and secrete functional enzymes, but that the enzyme activity levels differ significantly between strains.

### Glucuronoxylan growth assessment of engineered strains

**Fig. 3:**
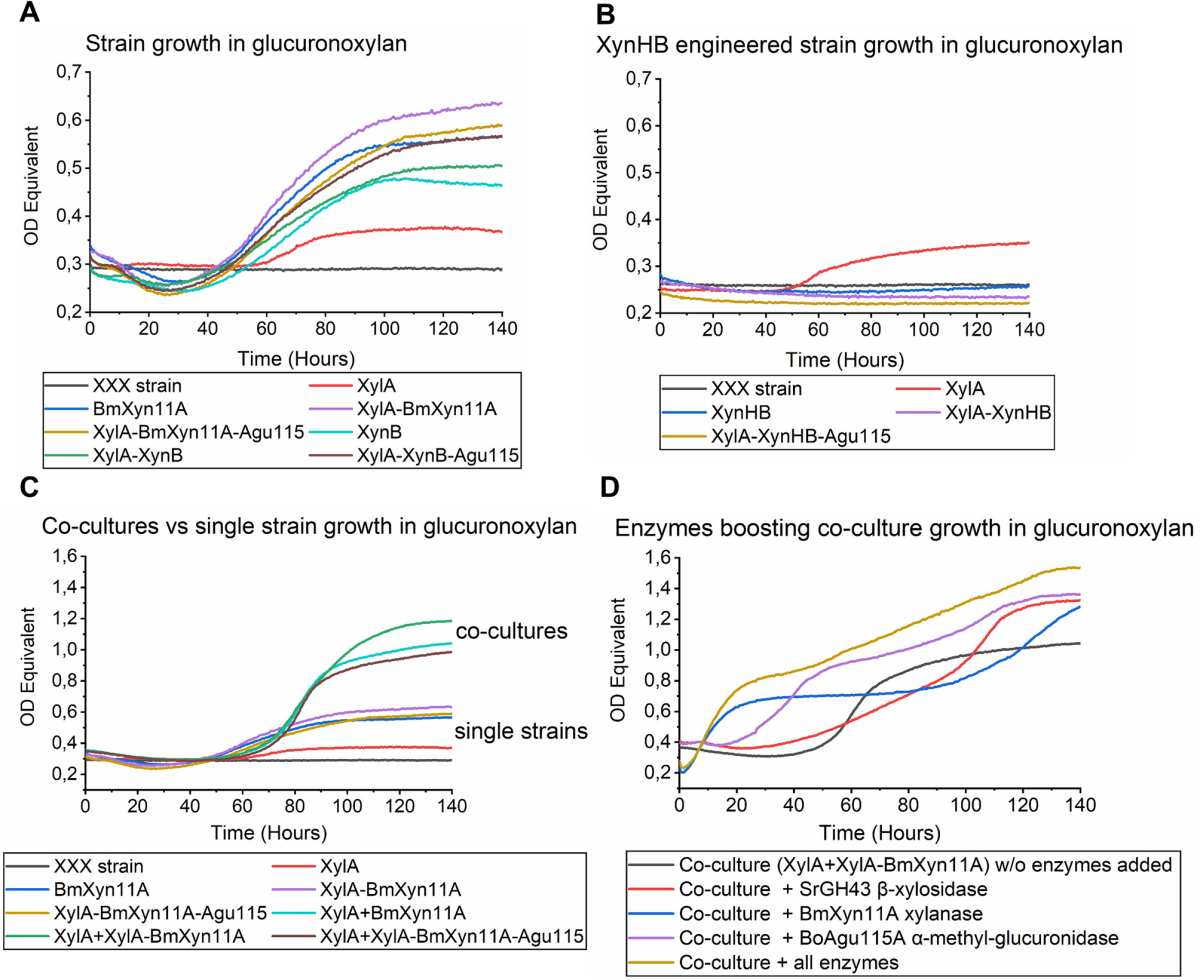
Yeast growth in beechwood glucuronoxylan. (**A**) Growth of CRISPR/Cas9 engineered strains with *Bm*Xyn11A or XynB xylanases compared to XXX and XylA strains in Delft medium with 20 g L^− 1^ beechwood GX. (**B**) Growth of XynHB engineered strains compared to XXX and XylA strains in beechwood GX. (**C**) Growth of single strains compared to co-cultures (ration 1:1) with XylA strain in beechwood GX. (**D**) Growth of co-culture XylA + XylA-BmXyn11A strains (ratio 1:1) when supplemented with exogenous xylanolytic enzymes (200 µg/g GX). All strains were grown in triplicates. OD Equivalent = Optical density normalized from *S. cerevisiae* growth in Delft/glucose medium in a Growth-Profiler 960. GX = glucuronoxylan.

Next, strains with confirmed recombinant xylanolytic activities were inoculated in medium with 20 g L^− 1^ beechwood GX and growth was followed over time. All strains engineered with *Bm*Xyn11A displayed an initial drop in OD_Equ_, indicating hydrolysis of the partly insoluble GX polysaccharide, followed by an increase in OD_Equ_ indicative of yeast growth (Fig. 3A). Doubling times and final OD_Equ_ values in Agu115-expressing strains were not improved compared to other strains, likely due to the poor secretion of the enzyme (Fig. 3A). Similar to what was observed in the enzymatic assays, strains expressing *Bm*Xyn11A showed shorter doubling times compared to strains expressing XynB (24.0-28.9 h versus 36.1–39.9 h, respectively) and also higher final OD_Equ_ (Fig. 3A). Interestingly, expression of XynHB xylanase did not manifest in yeast strain growth in liquid GX (Fig. 3B), even though the XynHB GH11 was shown to be expressed, secreted and active in the enzyme activity assays (Fig. 2B) and on agar plates (Fig. 2C). These results show the importance of coupling enzymatic activity with growth of the host strain, to ensure compatibility between the microorganism, the enzyme(s), and the hydrolysis products.

Due to the low XylA activity in strains expressing multiple enzymes, we combined selected strains in the same growth culture, hypothesizing that they would benefit from the other strain’s secreted enzymes and display a synergistic growth behavior. Indeed, co-cultures of the XylA strain and *Bm*Xyn11A-engineered strains (ratio 1:1) showed shorter doubling times (9.3–11.3 h) and higher final OD_Equ_ (1.0-1.2) than the respective monocultures with doubling times of 24.0-29.8 h and final OD_Equ_ of 0.5–0.7 (Fig. 3C). We also tested co-cultures with other starting ratios (1:10 and 10:1) (Supplementary Figure S3), but overall, the best performing co-culture was the XylA strain + XylA-BmXyn11A strain (ratio 1:1) reaching a final OD_Equ_ of 1.2, closely followed by XylA + BmXyn11A and the XylA + XylA-BmXyn11A-Aug115 with final OD_Equ_ of 1.0 (Fig. 3C). The 48 h long lag phase of the XylA + XylA-BmXyn11A co-culture could be greatly reduced with supplementation of *Bo*Agu115A at 200 µg/g GX (Fig. 3D), again suggesting that Agu115 is poorly expressed or secreted by the yeast. In fact, the addition of all enzymes, added individually or together, boosted growth of the XylA + XylA-BmXyn11A co-culture (Fig. 3D). Together, these results clearly show that the strains can readily hydrolyze and grow on GX, although further strain engineering aiming to optimize enzyme expression and secretion would likely improve strain performance.

### Co-culture fermentations of glucuronoxylan

**Fig. 4:**
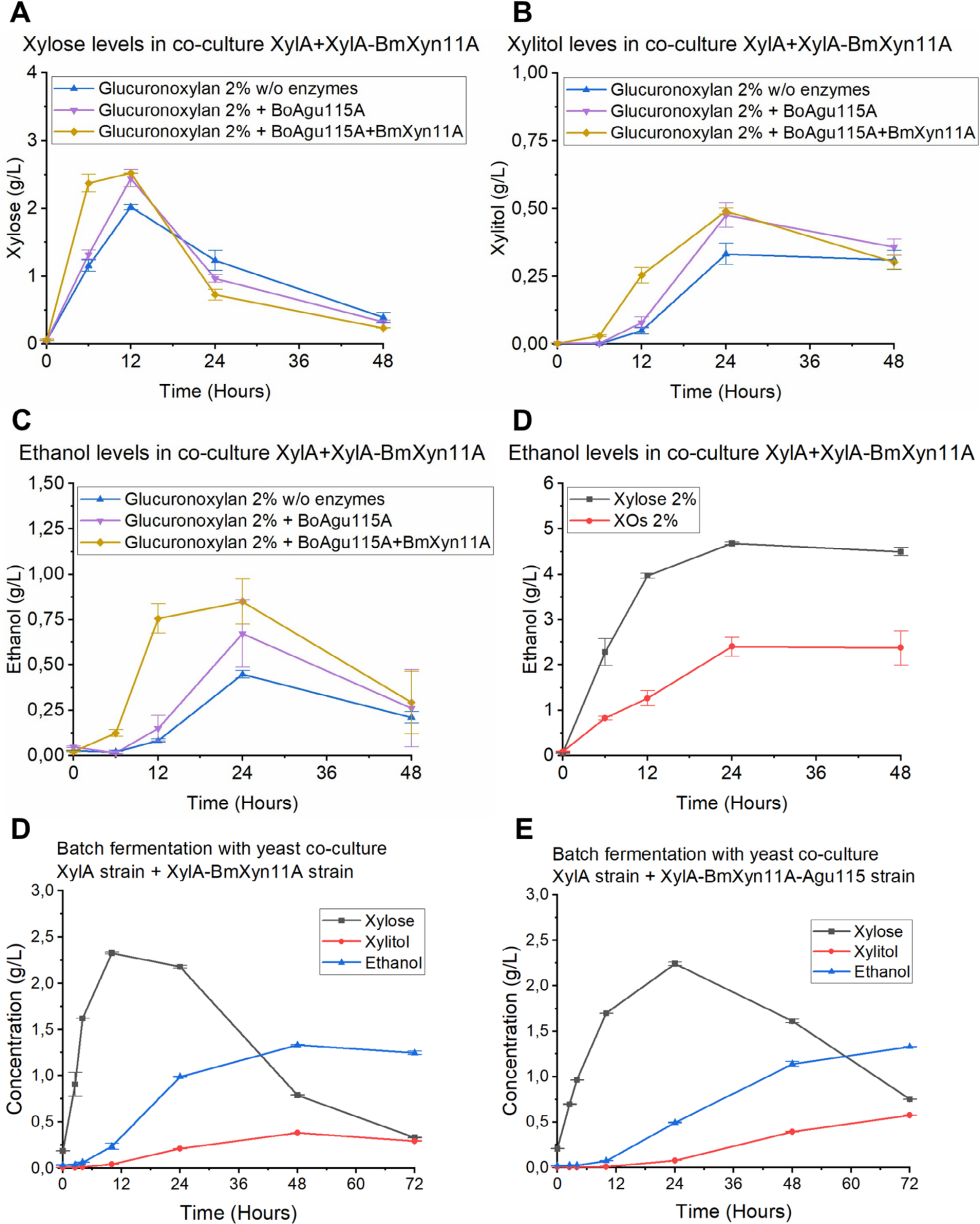
Co-culture fermentations of glucuronoxylan. (**A**) Xylose, (**B**) xylitol and (**C**) ethanol concentrations over time from 20 g L^− 1^ beechwood GX fermentation by co-culture of XylA strain + XylA-BmXyn11A strains (ratio 1:1) when supplemented with 100 µg/g GX of *Bm*Xyn11A and 100 µg/g GX of *Bo*Agu115A using flask fermentations with glycerol locks under oxygen limited conditions in triplicates. (**D**) ethanol concentrations over time from 20 g L^− 1^ xylose or XOs by co-culture of XylA strain + XylA-BmXyn11A strains (ratio 1:1). (**E**) DASGIP 300 mL batch fermentations of beechwood GX showing xylose consumption or xylitol and ethanol production over time comparing co-culture XylA strain + XylA-BmXyn11A strain and (**F**) co-culture XylA + XylA-BmXyn11A-Agu115 strain supplemented with 100 µg/g GX *Bm*Xyn11A and 50 µg/g GX *Bo*Agu115A. All co-cultures had an initial OD_600_ = 5 for each strain. GX = glucuronoxylan. XOs = xylooligosaccharides.

The best growing co-culture XylA + XylA-BmXyn11A was chosen for fermentation of beechwood GX under oxygen limited conditions, where the GX hydrolysis product xylose and the fermentation products xylitol and ethanol were analyzed over time. The fermentation tests were performed with and without externally added enzymes, to investigate the impact on enzyme supplementation on strain performance. During the fermentations, xylose was initially accumulated and then consumed in all co-cultures within a 48 h period (Fig. 4A), while xylitol (Fig. 4B) and ethanol (Fig. 4C) reached maximum titers after 24 h. Supplementation with α-methyl-glucuronidase *Bo*Agu115A and xylanase *Bm*Xyn11A to the XylA + XylA-BmXyn11A co-culture resulted in almost doubling in ethanol titer (0.85 g L^− 1^) compared cultures with no enzyme supplementation (0.45 g L^− 1^) after a 24 h period (Fig. 4C) corresponding to approximately 10% of the theoretical maximum ethanol titer that can be obtained from total xylose content in the beechwood GX medium. In comparison, co-cultures containing 2% xylose or 2% XOs (corncob) as carbon sources reached ethanol titers of 4.5 and 2.8 g L^− 1^, respectively (Fig. 4D) which correspond to approximately 44% and 27% of the theoretic maximum ethanol titers, respectively.

By instead using a 300 mL bioreactor batch fermentation setup supplemented with recombinant *Bm*Xyn11A and *Bo*Agu115A at 100 and 50 µg/g GX, respectively under well-controlled pH, temperature and O_2_ availability, ethanol titers could be further increased to 1.32 g L^− 1^ after a 48 h period in co-culture XylA + XylA-BmXyn11A (Fig. 4D) and to 1.33 g L^− 1^ after a 72 h period for co-culture XylA + XylA-BmXyn11A-Agu115 (Fig. 4E) corresponding to 15.1% of maximum theoretical ethanol yield. Overall, we can conclude that the strains engineered in this study display rapid xylitol and ethanol formation using GX as sole carbon source compared to previous studies, which often use incubation periods of > 100 h with similar ethanol yields (see Supplemental Table S1).

## Discussion

The objective of this study was to engineer *S. cerevisiae* with xylanolytic enzymes specifically targeting beechwood GX. Employing a targeted enzyme approach, we carefully matched hydrolytic enzyme activities to the chemical characteristics of the GX substrate. Further, we constructed a library of yeast strains, each expressing different combinations of enzymes, and could determine that the constructed strains expressed and/or secreted the enzymes and metabolized the GX hydrolysis products to different degrees. Notably, co-cultures of strains expressing complementary enzymes coupled with external enzyme supplementation boosted yeast growth and ethanol fermentation of GX.

As xylanases are central enzymes for GX degradation, a lot of our screening efforts focused on optimizing this activity in yeast. From the activity observed on xylan-coated cellulose thin films, the GH11 xylanase *Bm*Xyn11A showed better hydrolysis performance compared to the *Cj*GH10 and *Bo*GH30 xylanases tested in this study. Moreover, expression of different GH11 xylanases in yeast revealed that strains engineered with the *Bm*Xyn11A of yeast origin and XynB of fungal origin could grow on GX, while yeast expressing the bacterial XynHB xylanase could not. The reason(s) for this phenomenon is unclear but could be due to that XynHB produces xylooligosaccharides that are incompatible with the chosen XylA β-xylosidase, or that the xylanase was inhibited under the yeast culture conditions. In any case, the results showcase possible pitfalls and risks of combining enzyme systems from different donors into one heterologous host.

Whereas xylanase expression and secretion in *S. cerevisiae* worked well in our design, we observed that β-xylosidase expression and/or secretion was negatively affected when co-expressed with other heterologous enzymes. Moreover, strains seemed to struggle to express and/secrete the Agu115 α-methyl-glucuronidase. While further experimental investigation is needed to elucidate the cause of this, it is known that expression and secretion of heterologous proteins often places metabolic burdens on *S. cerevisiae*, especially if they are highly overexpressed [44]. Foreign proteins retained in the cytoplasm with suboptimal folding can lead to protein aggregation and an overload of unfolded or misfolded proteins in the lumen of the endoplasmic reticulum, causing secretion stress in the cell [42]. We speculate that such folding and secretion issues may have caused the low β-xylosidase and α-methyl-glucuronidase activities observed in our constructed strains, and where the smaller sized (∼ 21 kDa) xylanases seem to be favored for secretion in the co-expression strains. Future mitigation strategies to these issues include adaptive laboratory evolution to improve secretion characteristics and employing promoters of different strengths. Additionally, overexpression of Kar2p, Ssa1p, or PDI chaperone proteins can be used to enhance secretion of heterologous proteins [45], and the use of different secretion signals such as α-mating signal that circumvent the Golgi and relieve the Sed1 secretory pathway [46] can be attempted.

As co-expression of different enzymes in the same cell led to decreased extracellular enzyme activities, we instead designed co-culture systems. Here, we saw a clear synergistic growth advantage of yeast co-cultures engineered with the *Bm*Xyn11A xylanase compared to single strains, as judged by doubling times of 9.3–11.3 h in GX-containing growth medium compared to monocultures with doubling times of 24.0-29.8 h. In such co-cultures, the engineered yeast strains ideally share the heterologous enzyme expression and secretion burden and collaborate in a synergistic manner to hydrolysis GX to benefit equally from the released monosaccharides. This is also a common strategy in nature, where microbes are known to rely on xylan degradation from other species’ hydrolytic capacities [22, 47]. In our study, a limited number of different yeast co-culture ratios were tested (1:10, 1:1, 10:1), and we found that the yeast starting ratio of 1:1 resulted in most efficient xylan growth. Co-culture fermentations also resulted in ethanol titers that were comparable to previous findings using xylans as sole carbon source, although we used a relatively low starting OD = 5 per strain and reached the maximum titers faster than strains in other studies (Supplemental Table S1). In the future, synthetic yeast consortium systems could be developed with several different strains and yeast ratios optimized for the hydrolysis of specific biomasses and their chemical composition [48].

In this study, heterologous xylanolytic enzymes were targeted for secretion to the extracellular space, as this has been shown advantageous for producing hydrolytic enzymes at high levels [49]. However, secretion makes identification and selection of superior enzyme producing strains difficult, as the enzymes are not tethered to the cells. This can also be a disadvantage in industrial applications, where enzymes produced in pre-cultures risk being lost during yeast harvest procedures. In line with this, our xylanolytic engineered strains displayed a long lag phase (48 h) in the beechwood GX cultures, likely due to the need of synthesizing new heterologous enzymes to facilitate growth. This can be remedied by supplementation of recombinant enzymes, or by transferring the pre-culture supernatant along with the yeast cells to the experimental vessel. Another alternative is to display enzymes on the cell surface [50], which is an approach used by many natural xylanolytic yeasts [7]. However, the amount of space on a single yeast cell surface is limited and excess enzyme production may lead to cell metabolism imbalances, ultimately lowering the total amount of expressed and secreted enzymes [51].

## Conclusions and outlook

In this work, the xylose-fermenting *S. cerevisiae* CEN.PK XXX strain was engineered using CRISPR/Cas9 genomic editing technology for expression of enzymes specifically targeting GX. Out of a large number of different strains constructed, the best GX-converting strains expressed a yeast-derived *Bm*Xyn11A xylanase in combination with a fungal XylA β-xylosidase. Further, *S. cerevisiae* GX growth was clearly improved by adding an α-methyl-glucuronidase that removes 4-*O*-methyl-D-glucuronic acid sidechains from the GX backbone, which to our knowledge has not been studied previously. In the future, expression of other debranching enzymes such as acetyl xylan esterases or arabinofuranosidases [23] can be assessed, as well as combining different xylanases to further hydrolyze GX, such as GH5 xylanases or GH30_7 glucuronoxylanases that target the GX backbone at the 4-*O*-methyl-D-glucuronic acid sidechains [52, 53]. While expression and secretion of multiple heterologous enzymes proved to be challenging in *S. cerevisiae*, this issue can be solved by co-culturing of engineered *S. cerevisiae* strains expressing different xylanolytic enzymes. Collectively, the results presented expand our current knowledge of strain engineering for GX hydrolysis and fermentation. The developed strains have large potential for future use in industrial GX-based bioprocesses. They can also be used as screening platforms for testing different combinations of xylanolytic enzymes and co-culture design, to further optimize GX hydrolysis and fermentation.

### Electronic supplementary material

Below is the link to the electronic supplementary material.

**Supplementary Material 1:** MOCLO cloning strategy

**Supplementary Material 2: Fig. S2.** Strain development and colony PCR confirmation of genomic integration of recombinant xylanolytic genes into the CEN.PK XXX strain

**Supplementary Material 3: Fig. S3.** Growth of co-cultures XylA+XylA-BmXyn11A and XylA+BmXyn11A strains in 2% beechwood glucuronoxylan at different strain ratios (1:1, 10:1 and 1:10)

**Supplementary Material 4: Table S2.** Plasmids applied to assemble plasmids in Table 1 and primers to check assembly and integration into the genomic X2 site

**Supplementary Material 5: Table 1.** Ethanol titers produced from xylan fermentation by recombinant *S. cerevisiae* strains

## Data Availability

No datasets were generated or analysed during the current study.

## Abbreviations

CE: Carbohydrate esterase
CRISPR: Clustered Regularly Interspaced Short Palindromic Repeats
DNS: Dinitro salicylate
GH: Glycoside hydrolase
GX: Glucuronoxylan
SPR: Surface plasmon resonance spectroscopy
SSF: Simultaneous saccharification and fermentation
