# Supplementary material for: Engineering Saccharomyces cerevisiae for targeted hydrolysis and fermentation of glucuronoxylan through CRISPR/Cas9 genome editing
Source: Microb Cell Fact. 2024 Mar 16;23:85. doi: 10.1186/s12934-024-02361-w (PMC10943827; doi:10.1186/s12934-024-02361-w)
Supplement: Supplementary file 2 — Supplementary Material 2: Fig. S2. Strain development and colony PCR confirmation of genomic integration of recombinant xylanolytic genes into the CEN.PK XXX strain [file 12934_2024_2361_MOESM2_ESM.docx]

**Supplemental Figure S2.** Strain development and colony PCR confirmation of genomic integration of recombinant xylanolytic genes into the CEN.PK XXX strain.


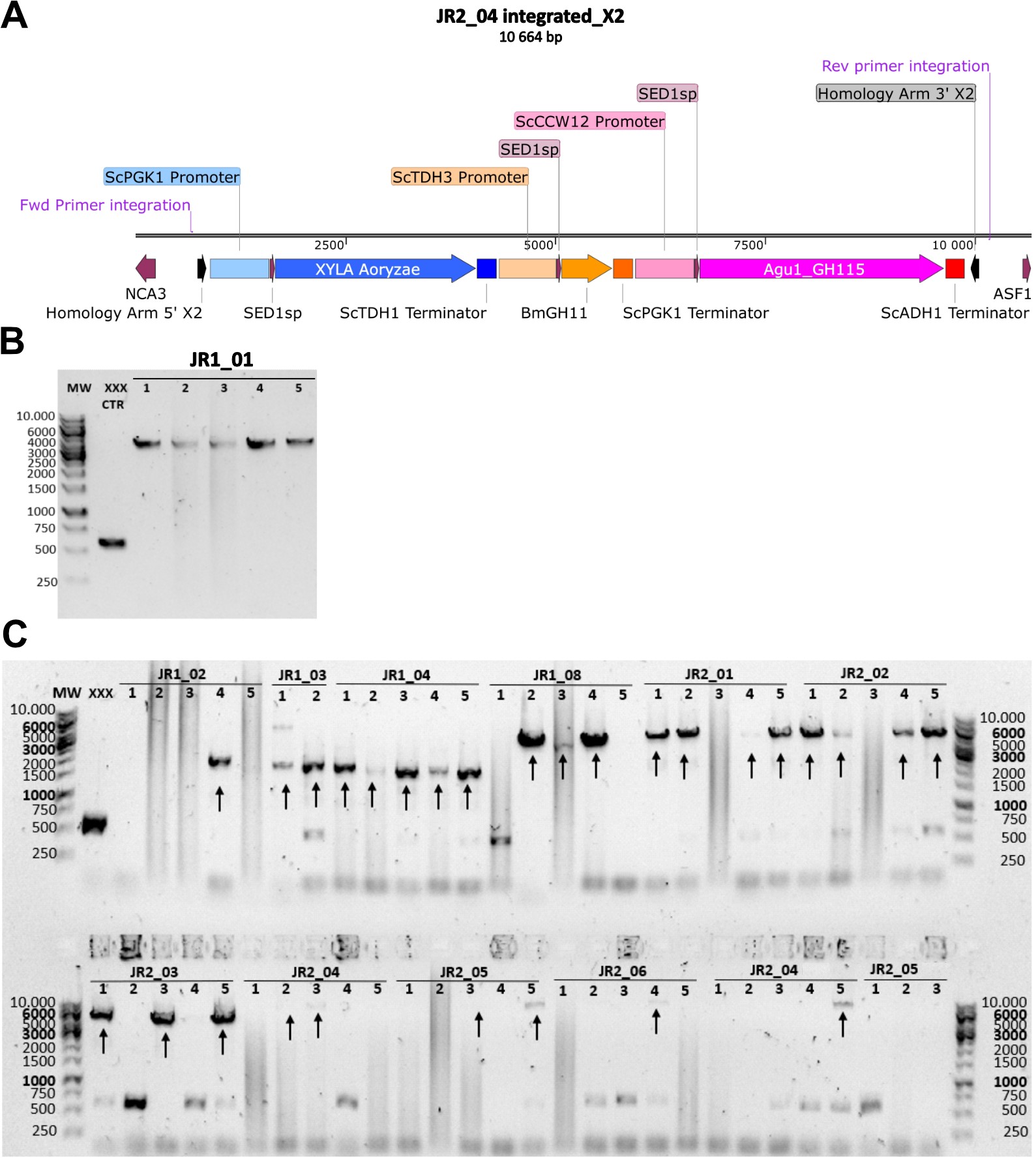
**Supplementary Figure S2. Genomic integration of genes by CRISPR/Cas9.** (A) Schematic map of the X2 locus situated between the NCA3 and ASF1 genes in *S. cerevisiae* chromosome X with successful integrated recombinant XylA β-xylosidase, *Bm*Xyn11A xylanase and Agu115 α-methyl-glucuronidase genes after homologous recombination of the Homology arms. The pJR2_04_SED1-XylA-BmXyn11A-Agu115 plasmid is linearized by NotI prior to homologous recombination at the Cas9 mediated double stranded break. (B) Colony PCR gel showing successful integration of XylA gene (4220 bp) into the XXX strain using integration forward primer LTR183 and reverse primer LTR182R aligning to genome sequence outside the integration X2 site to construct the XylA strain. No integration is indicated by a band at 590 bp. (C) Successful integration of single (~2404 bp for xylanases or 4734 bp for Agu115), double (XylA with xylanases ~5587 bp) and triple (~10670 bp) gene integration indicated by black arrows. A single band indicates integration in each allele in the diploid *S. cerevisiae*.
