# Supplementary material for: Engineering Saccharomyces cerevisiae for targeted hydrolysis and fermentation of glucuronoxylan through CRISPR/Cas9 genome editing
Source: Microb Cell Fact. 2024 Mar 16;23:85. doi: 10.1186/s12934-024-02361-w (PMC10943827; doi:10.1186/s12934-024-02361-w)

**Supplemental Figure S3.** Growth of co-cultures XylA+XylA-BmXyn11A and XylA+BmXyn11A strains in 2 % beechwood glucuronoxylan at different strain ratios (1:1, 10:1 and 1:10).


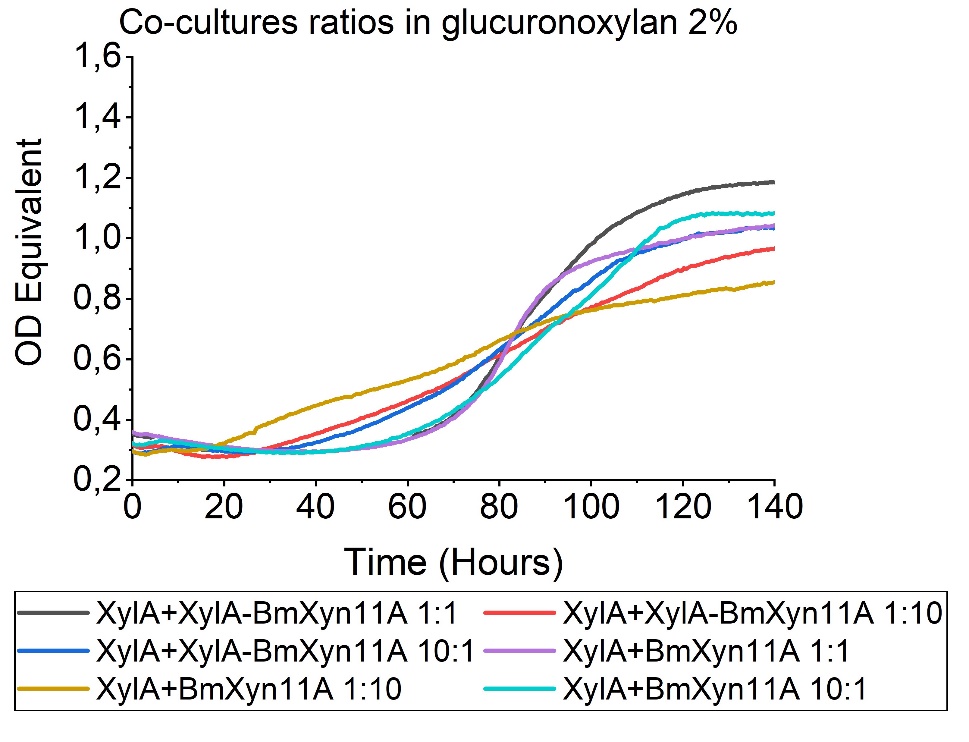

Supplement: Supplementary file 3 — Supplementary Material 3: Fig. S3. Growth of co-cultures XylA+XylA-BmXyn11A and XylA+BmXyn11A strains in 2% beechwood glucuronoxylan at different strain ratios (1:1, 10:1 and 1:10) [file 12934_2024_2361_MOESM3_ESM.docx]
