# Supplementary material for: Engineering Saccharomyces cerevisiae for targeted hydrolysis and fermentation of glucuronoxylan through CRISPR/Cas9 genome editing
Source: Microb Cell Fact. 2024 Mar 16;23:85. doi: 10.1186/s12934-024-02361-w (PMC10943827; doi:10.1186/s12934-024-02361-w)
Supplement: Supplementary file 4 — Supplementary Material 4: Table S2. Plasmids applied to assemble plasmids in Table 1 and primers to check assembly and integration into the genomic X2 site [file 12934_2024_2361_MOESM4_ESM.docx]

**Supplementary Table S2.** Plasmids applied to assemble plasmids in Table 1 and primers to check assembly and integration into the genomic X2 site.

| **Plasmid** | **Description** | **Source** |
| --- | --- | --- |
| pYTK001 | Entry plasmids backbone containing GFP gene dropout and CamR resistance gene. | Lee et al. 2015 |
| pYTK002-004 | Plasmids containing assembly connectors ConLS, ConL1, ConL2, respectively. | Lee et al. 2015 |
| pYTK009-011 | Plasmids containing promoters *Sc*TDH3, *Ss*CCW12 or *Sc*PGK1, respectively. | Lee et al. 2015 |
| pYTK053-056 | Plasmids containing terminators *Sc*ADH1, *Sc*PGK1 and *Sc*TDH1, respectively. | Lee et al. 2015 |
| pYTK067-068 and 072 | Plasmids containing assembly connectors ConR1, ConE, ConR2, respectively. | Lee et al. 2015 |
| SED1 | *SED1* signal peptide (57 bp) was cloned into MoClo’s pYTK001 as part 3a (N-terminal CDS)  SED1 sequence: ATGAAATTGTCTACTGTTTTGTTATCAGCTGGTTTGGCATCTACTACATTAGCTCAA | This study |
| pJR_1_01_SED1-XylA | Plasmid containing XylA CDS. The codon optimized CDS is found in supplementary File 1 sheet 2. CDS was adapted as type 3b. | This study |
| pJR1_02_SED1-BmXyn11A | Plasmid containing BmXyn11A CDS and ConL1 -ConRE assembly connectors. The codon optimized CDS is found in supplementary File 1 sheet 2. CDS was adapted as type 3b from vector BmGH11_pUC57-Kan from GeneScript (USA). | This study |
| pJR1_03_SED1-XynHB | Plasmid containing XynHB CDS.  The codon optimized CDS is found in supplementary File 1 sheet 2. CDS was adapted as type 3b from vector XynHB_pUC57-Kan from GeneScript (USA). | This study |
| pJR1_04_SED1-XynB | Plasmid containing XynB CDS.  The codon optimized CDS is found in supplementary File 1 sheet 2. CDS was adapted as type 3b from vector XynB_pUC57-Kan from GeneScript (USA). | This study |
| pJR1_05_SED1-BmXyn11A | Plasmid containing BmXyn11A CDS for multigene insert. The codon optimized CDS is found in supplementary File 1 sheet 2. CDS was adapted as type 3b from vector BmGH11_pUC57-Kan from GeneScript (USA). | This study |
| pJR1_06_SED1-XynHB | Plasmid containing XynHB CDS for multigene insert. The codon optimized CDS is found in supplementary File 1 sheet 2. CDS was adapted as type 3b from vector XynHB_pUC57-Kan from GeneScript (USA). | This study |
| pJR1_06_SED1-XynB | Plasmid containing XynB CDS for multigene insert. The codon optimized CDS is found in supplementary File 1 sheet 2. CDS was adapted as type 3b from vector XynB_pUC57-Kan from GeneScript (USA). | This study |
| pJR1_08_SED1-Agu115 | Plasmid containing Agu115 CDS for multigene insert. The codon optimized CDS is found in supplementary File 1 sheet 2. CDS was adapted as type 3b from vector Agu115_pUC57-Kan from GeneScript (USA). | This study |
| pJR2_01_SED1-XylA-BmXyn11A | Plasmid containing XylA and BmXyn11A CDS. The codon optimized CDS is found in supplementary File 1 sheet 2. Multigene construct was assembled by connectors ConL1 and ConR2. | This study |
| pJR2_02_SED1-XylA-XynHB | Plasmid containing XylA and XynHB CDS. The codon optimized CDS is found in supplementary File 1 sheet 2. Multigene construct was assembled by connectors ConL1 and ConR2. | This study |
| pJR2_03_SED1-XylA-XynB | Plasmid containing XylA and XynB CDS. The codon optimized CDS is found in supplementary File 1 sheet 2. Multigene construct was assembled by connectors ConL1 and ConR2. | This study |
| pJR2_04_SED1-XylA-BmXyn11A-Agu115 | Plasmid containing XylA, BmXyn11A and Agu115 CDS. The codon optimized CDS is found in supplementary File 1 sheet 2. Multigene construct was assembled by connectors ConL1, ConL2, ConR2 and ConRE. | This study |
| pJR2_05_SED1-XylA-XynHB-Agu115 | Plasmid containing XylA, XynHB and Agu115 CDS. The codon optimized CDS is found in supplementary File 1 sheet 2. Multigene construct was assembled by connectors ConL1, ConL2, ConR2 and ConRE. | This study |
| pJR2_06_SED1-XylA-XynB-Agu115 | Plasmid containing XylA, XynB and Agu115 CDS. The codon optimized CDS is found in supplementary File 1 sheet 2. Multigene construct was assembled by connectors ConL1, ConL2, ConR2 and ConRE. | This study |
| LT1_30_backbone_X2_integration | Multi Backbone for the X2 locus | Addgene Kit # #1000000215  Torello Pianale and Olsson 2023 |
| LT1_63-66_backbone_X2_integration | Transcriptional units (TU1-3) backbones for multigene insertion and homology arms for genomic integration at the X2 locus. | Addgene Kit # #1000000215 Torello Pianale and Olsson 2023 |
| pYN2_1 | Guide RNA scaffold plasmid targeting the Chromosome 10 intergenic region gRNA scaffold combination with cas9. Contains the G418 selection marker | Addgene #184757  Cámara, Lenitz, and Nygård 2020 |
| pYN2_1_LT58 | Cas9 plasmid with sgRNA cassette for integration in the X2 locus in chromosome 10 | Addgene #177705  Torello Pianale and Olsson 2023 |
| **Primers** | **Oligonucleotide sequence (5’-3’)** | **Application** |
| LT126_LocusX2_check F | TTGCCGTCAAAAGATCCTCTCATAC | TU assembly level 1, 2 |
| LT126_LocusX2_check R | CGGCTTCTCATAAAACGTCCCAC | TU assembly level 1, 2 |
| LT183_LocusX2_#4_F | TGCTCGATCTTCTATCCTCTTTAGG | Genomic integration X2 site |
| LT182_LocusX2_#3_R | GTGAGGACAGGCTTAATTGAGC | Genomic integration X2 site |
