# Supplementary material for: Engineering Saccharomyces cerevisiae for targeted hydrolysis and fermentation of glucuronoxylan through CRISPR/Cas9 genome editing
Source: Microb Cell Fact. 2024 Mar 16;23:85. doi: 10.1186/s12934-024-02361-w (PMC10943827; doi:10.1186/s12934-024-02361-w)
Supplement: Supplementary file 5 — Supplementary Material 5: Table 1. Ethanol titers produced from xylan fermentation by recombinant S. cerevisiae strains [file 12934_2024_2361_MOESM5_ESM.docx]

**Supplemental Table 1.** Ethanol titers produced from xylan fermentation by recombinant *S. cerevisiae* strains.

| **Strain name** | **Base yeast strain/genotype** | **Ploidy** | **Genotype** | **Cultivation conditions*** | **Fermentation time (h)** | **Target product (TP)** | **Titers of TP** | **Yield of TP** | **Ref.** |
| --- | --- | --- | --- | --- | --- | --- | --- | --- | --- |
| MT8-1/pUCSXIIXA/pWX1X2XK | MT8-1/  MATa ade his3 leu2 trp1 ura3 | N | **Replicative plasmid** carrying *XYNII* from *T. reesei* and *XlnD* from *A. niger* | SC medium supplemented with **100 g L^-1^ of** **birchwood xylan.** An oxygen-limited condition was applied (closed bottles equipped with a bubbling CO_2_ outlet). **Initial OD: 50.** | 62 h | Ethanol | 7.1 g L^-1^ | 0.30 g g^-1^ | Katahira et al., 2004 |
| OC2-AXYL2-ABGL2-Xyl2 | OC2-HUT/  Mata/α his3/his3 ura3/ura3 trp1/trp1 | 2N | **Integration** of *XylA* from *A. oryzae* | YPKX medium with 40 g L^-1^ **cellulose (KC-flock)** and **40 g L^-1^ xylan from Birchwood** as carbon sources. 30 g L^-1^ cellulase was added. **Initial OD: 6.** | 48 h | Ethanol | 12.5 g L^-1^ | 0.52 g g^-1^ (total) | Saitoh et al., 2011 |
| D-XSD/XKXDHXR | D452-2/  MATa leu2 his3 ura3 can1 | N | **Replicative plasmid** carrying *bxl1* from *T. reesei*. | SC medium with **40 g L^-1^** **XOS** in closed bottles. **Initial OD: 20.** | 168 h | Ethanol | 4.2 g L^-1^ | NS | Fujii et al., 2011 |
| HZ3345 | L2612/  MATα leu2-3 leu2-112 ura3-52 trp1-298 can1 cyn1 gal+ | N | **Replicative plasmid** carrying *XynII* from *T. reesei* and *XlnD* from *A. niger* | Fermentation medium with 10 g L^-1^ yeast extract, 20 g L^-1^ peptone, **10 g L^-1^** **birchwood xylan**, 0.001% ergosterol, and 0.042% Tween 80. The cultivation was performed anaerobically in serum bottles. **Initial OD: 50.** | 80 h | Ethanol | 0.95 g L^-1^ | 0.31 g g^-1^ | Sun et al., 2012 |
| Sc-K2 | INVSc1/  MATa his3D1 leu2 trp1-289 ura3-52 MATα his3D1 leu2 trp1-289 ura3-52 | 2N | **Replicative plasmid** carrying *XYN* and *XYL* from *A. terreus* | Fed-batch in shake flasks with 20 g L^-1^ **glucose**, 20 g L^-1^ tryptone, 10 g L^-1^ yeast extract, **3 g L^-1^ xylan. Initial OD: 0.5.** | 168 h | Xylitol | 1.94 g L^-1^ | 0.71 g g^-1^ | Li et al., 2013 |
| NS | L2612/  MATα leu2-3 leu2-112 ura3-52 trp1-298 can1 cyn1 gal+ | N | **Replicative plasmid** carrying *XNA1, XD2 and ABF* from *Ustilago bevomyces.* An ALE approach was applied to improve xylan utilization. | Complex (YPXN) or minimal medium (CSM) with **20 g L^-1^ of** **beechwood xylan**. An oxygen-limited condition was applied (in a sealed 14-mL culture tube containing 5 mL of medium). **Initial OD: 20.** | 120 h | Ethanol | YPXN: 0.32 g L^-1^  CSM: 0.22 g L^-1^ | NS | Lee et al., 2015 |
| Y294[YMX1] | Y294/  MATα leu2-3,112 ura3-52 his3 trp1-299 | N | **Replicative plasmid** carrying *Xyn2* from *T. reesei* and *XlnD* from *A. Niger*. | YPBX medium (10 g L^-1^ yeast extract, 20 g L^-1^ peptone, **50 g L^-1^ of Beechwood**). The cultivation was performed aerobically in shake flasks. **Initial OD:** **10 % (v/v) preculture.** | 28 days | Biomass | NS | NS | Mert et al., 2016 |
| BYs-Xyn11B_XlnD | BY4741/ Δsed1/  MATa, his3 Δ1, leu2 Δ0, met15 Δ0, ura3 Δ 0, *YDR077w::kanMX4* | N | **Replicative plasmid** carrying *Xyn11B* from *Saccharophagus degradans* and *XlnD* from *A. Niger*. | Co-culture (OD50 for each strain) of two strains: one co-displays Xyn11B and XlnD and a D-xylose-isomerizing strain that displays XylC in SC medium (SXNC) with **100 g L^-1^** **birchwood xylan**, 2 g L^-1^ casamino acids, 100 mM MES (pH 6.7), and 3 mM cobalt(II) chloride. An oxygen-limited condition was applied (closed tubes). **Initial OD: 100.** | 200 h | Ethanol | 6.0 g L^-1^ | NS | Sasaki et al., 2017 |
| Consortium of engineered xylose-utilizing strains expressing different hemicellulases | EBY100/  MATa AGA1::GAL1 -AGA1::URA3 ura3 -52 trp1 leu2 -delta200 his3 -delta200 pep4::HIS3 prb11.6R can1 GAL | N | **Replicative plasmid** contaning a set of different hemicellulase encoding genes including *Abf1*, *Axe1*, *Bxl1*, *Glr1*, and *Xyn2* from *T. reesei*. The hemicellulases were displayed in the cell surface. | SC with **10 g L^-1^** **Beechwood Xylan** or **20 g L^-1^** **Wheat Arabinoxylan** buffered with 50 mM Na-citrate (∼pH 6) containing 12 mM CaCl 2 and 2 mM EDTA. **Initial OD: ∼3.0–5.0.** | 168 h | Ethanol | Beechwood Xylan: 0.96 g L^-1^  Wheat Arabinoxylan:0.778 g L^-1^ | Beechwood Xylan:  Wheat Arabinoxylan: | Tabañag et al., 2018 |
| BSGIBX | CEN.PK 113-5D/ MATa ura3-52 HIS3, LEU2 TRP1 MAL2-8c SUC2 | N | **Replicative plasmid** carrying *Xyl3A* from *P. Oxalicum*. | SC medium with **20 g L^-1^** **XOS** with and without treatment with xylanase (mg g^-1^ XOS) as the carbon source. An oxygen-limited condition was applied (serum bottles with a rubber stopper plug). **Initial OD: 0.5 g L^-1^.** | 60 h | Ethanol | Without treatment: 4.37 g L^-1^  Treated: 9 g L^-1^ | NS | Niu et al., 2019 |
| M-GH43-S-x2 | S288c/  MATα SUC2 mal mel gal2 CUP1 flo1 flo8-1 hap1 | N | **Integration** of *xln43* from *Pyrenophora tritici-repentis* and *Xyn2* from *T. reesei*. The beta-xylosidade (GH43) was displayed in the cell surface. | YP medium with **20 g L^-1^**  **beechwood xylan**. An oxygen-limited condition was applied (glass bottles with a rubber stopper plug). **Initial OD: 1.** | 120h | Ethanol | 0.47 g L^-1^ | NS | Kruger & den Haan, 2022 |

NS: Not specified. *SC medium: 0.67% yeast nitrogen base supplemented with amino acids. YP medium: 1% Yeast extract and 2% Peptone

**References:**

Fujii, T., Yu, G., Matsushika, A., Kurita, A., Yano, S., Murakami, K., & Sawayama, S. (2011). Ethanol production from xylo-oligosaccharides by xylose-fermenting Saccharomyces cerevisiae expressing β-xylosidase. Bioscience, Biotechnology and Biochemistry, 75(6), 1140–1146. https://doi.org/10.1271/bbb.110043

Katahira, S., Fujita, Y., Mizuike, A., Fukuda, H., & Kondo, A. (2004). Construction of a xylan-fermenting yeast strain through codisplay of xylanolytic enzymes on the surface of xylose-utilizing Saccharomyces cerevisiae cells. Applied and Environmental Microbiology, 70(9), 5407–5414. https://doi.org/10.1128/AEM.70.9.5407-5414.2004

Kruger, F., & den Haan, R. (2022). Surface tethered xylosidase activity improved xylan conversion in engineered strains of Saccharomyces cerevisiae. Journal of Chemical Technology and Biotechnology, 97(5), 1099–1111. https://doi.org/10.1002/jctb.7044

Lee, S. M., Jellison, T., & Alper, H. S. (2015). Xylan catabolism is improved by blending bioprospecting and metabolic pathway engineering in Saccharomyces cerevisiae. Biotechnology Journal, 10(4), 575–575. https://doi.org/10.1002/biot.201400622

Li, Z., Qu, H., Li, C., & Zhou, X. (2013). Direct and efficient xylitol production from xylan by Saccharomyces cerevisiae through transcriptional level and fermentation processing optimizations. Bioresource Technology, 149, 413–419. https://doi.org/10.1016/j.biortech.2013.09.101

Mert, M. J., la Grange, D. C., Rose, S. H., & van Zyl, W. H. (2016). Engineering of Saccharomyces cerevisiae to utilize xylan as a sole carbohydrate source by co-expression of an endoxylanase, xylosidase and a bacterial xylose isomerase. Journal of Industrial Microbiology and Biotechnology, 43(4), 431–440. https://doi.org/10.1007/s10295-015-1727-1

Niu, Y., Wu, L., Shen, Y., Zhao, J., Zhang, J., Yi, Y., Li, H., & Bao, X. (2019). Coexpression of β-xylosidase and xylose isomerase in Saccharomyces cerevisiae improves the efficiency of saccharification and fermentation from xylo-oligosaccharides. Cellulose, 26(13–14), 7923–7937. https://doi.org/10.1007/s10570-019-02650-3

Saitoh, S., Tanaka, T., & Kondo, A. (2011). Co-fermentation of cellulose/xylan using engineered industrial yeast strain OC-2 displaying both β-glucosidase and β-xylosidase. Applied Microbiology and Biotechnology, 91(6), 1553–1559. https://doi.org/10.1007/s00253-011-3357-5

Sasaki, Y., Takagi, T., Motone, K., Kuroda, K., & Ueda, M. (2017). Enhanced direct ethanol production by cofactor optimization of cell surface-displayed xylose isomerase in yeast. Biotechnology Progress, 33(4), 1068–1076. https://doi.org/10.1002/btpr.2478

Sun, J., Wen, F., Si, T., Xu, J. H., & Zhao, H. (2012). Direct conversion of xylan to ethanol by recombinant Saccharomyces cerevisiae strains displaying an engineered minihemicellulosome. Applied and Environmental Microbiology, 78(11), 3837–3845. https://doi.org/10.1128/AEM.07679-11

Tabañag, I. D. F., Chu, I. M., Wei, Y. H., & Tsai, S. L. (2018). Ethanol production from hemicellulose by a consortium of different genetically-modified sacharomyces cerevisiae. Journal of the Taiwan Institute of Chemical Engineers, 89, 15–25. https://doi.org/10.1016/j.jtice.2018.04.029
